# Cellular Immunotherapy Targeting Cancer Stem Cells: Preclinical Evidence and Clinical Perspective

**DOI:** 10.3390/cells10030543

**Published:** 2021-03-04

**Authors:** Chiara Donini, Ramona Rotolo, Alessia Proment, Massimo Aglietta, Dario Sangiolo, Valeria Leuci

**Affiliations:** 1 Department of Oncology, University of Turin, 10124 Turin, Italy; chiara.donini@unito.it (C.D.); alessia.proment@edu.unito.it (A.P.); massimo.aglietta@unito.it (M.A.); 2 Candiolo Cancer Institute, FPO–IRCCS, Str. Prov. 142, km 3,95, 10060 Candiolo (TO), Italy; ramona.rotolo@ircc.it (R.R.); valeria.leuci@ircc.it (V.L.)

**Keywords:** cancer stem cells (CSCs), immunotherapy, adoptive immunotherapy, chimeric antigen receptor (CAR)

## Abstract

The term “cancer stem cells” (CSCs) commonly refers to a subset of tumor cells endowed with stemness features, potentially involved in chemo-resistance and disease relapses. CSCs may present peculiar immunogenic features influencing their homeostasis within the tumor microenvironment. The susceptibility of CSCs to recognition and targeting by the immune system is a relevant issue and matter of investigation, especially considering the multiple emerging immunotherapy strategies. Adoptive cellular immunotherapies, especially those strategies encompassing the genetic redirection with chimeric antigen receptors (CAR), hold relevant promise in several tumor settings and might in theory provide opportunities for selective elimination of CSC subsets. Initial dedicated preclinical studies are supporting the potential targeting of CSCs by cellular immunotherapies, indirect evidence from clinical studies may be derived and new studies are ongoing. Here we review the main issues related to the putative immunogenicity of CSCs, focusing on and highlighting the existing evidence and opportunities for cellular immunotherapy approaches with T and non-T antitumor lymphocytes.

## 1. Introduction

The term cancer stem cell (CSC) dates from about 25 years ago [1], with the evidence of a small population of stem-like cancer cells. CSCs are indeed a subpopulation of tumor cells considered responsible for tumorigenesis, metastasis and disease recurrence. CSCs apparently share key biological features with normal stem cells, such as the multi-differentiation ability and self-renewal capacity [2], but these properties are abnormally activated in CSCs. The concept and definition itself of CSCs are somehow not univocally defined [3]. In human cancers many attempts have been proposed to define CSCs through either different surface antigen expression patterns [4,5], transcription factors [6], signaling pathways or functional features [7,8,9]. Such “identity” issues are more prominent in the field of solid tumors while appear less present for. With the deepening of tumor biology research, multiple solid tumors have been found to be more clearly driven by CSCs compared with others, such as breast cancer [5], glioblastoma [10], prostate cancer [11], lung cancer [12], colorectal cancer, gastric cancer and liver cancer [13]. In other tumors, such as melanoma, the existence of a CSC compartment has been advocated [14] but also disputed by functional evidence supporting the high tumorigenicity of multiple melanoma cells irrespectively of the supposed CSC markers [7]. Therefore, there is an urgent need to identify reliable antigens to distinguish CSCs. At this aim, even antigens associated with enrichment of CSCs may be effectively useful [15].

CSCs represent one of the main obstacles in tumor treatment, because they can resist most of standard therapies (e.g., chemotherapy, radiotherapy, and molecularly targeted drugs) [16,17,18,19]. Cancer patients usually suffer from relapse and cancer recurrence may be due to CSCs resistance, differentiation ability and capacity of initiating new tumors after treatments [20,21].

CSCs may present multiple strategies to circumvent the immune attack, including genetic and non-genetic alterations that allow reduced immune recognition, enhanced tolerance to cytotoxic effects of immunity and promotion of a protective immunosuppressive tumor microenvironment (TME) [22]. The TME can evolve as the tumor progresses and various components participate to create a hypoxic, inflammatory, and immunosuppressive environment that facilitates tumor growth, progression and preservation of CSCs [23,24]. Multiple therapeutic approaches have been designed with the aim of killing CSCs and altering the TME. Some of these strategies are under evaluation in preclinical and clinical studies [25]. In recent years, cell-based immunotherapy has achieved promising outcomes in treating various malignancies. Here we focus on immunogenic properties of CSCs in solid tumors and review how CSCs may be targeted with immunological approaches based on killer lymphocytes. The heterogeneity of CSCs is so complex that surface antigens associated with enrichment of CSCs have been effectively useful also to target CSCs [3,26,27] as it is likely that some CSC antigens may be expressed also in non-CSCs, providing opportunities for enhanced immunotherapy [15,28].

We will discuss two distinct main strategies based on effector cells belonging to the adaptive immune system or to innate immune response. Furthermore, we will describe for each of them the successful preclinical and clinical outcomes, specifically focusing on results reached with the genetic engineering strategy of chimeric antigen receptor (CAR).

## 2. Identification and Immunological Properties of CSCs

In order to prevent or significantly delay relapse, CSCs should be specifically targeted and eliminated. CSCs may be identified based on immunological characteristics, on alterations of stem cell signaling pathways and on specific CSC markers and tumor associated antigens (TAA) [29]. 

### 2.1. Immunological Features

Several preclinical studies showed that CSCs are characterized by low immunogenicity and their immunological features can dictate an immunosuppressive activity. Immune evasion has been identified as an intrinsic property of CSCs, capable to modulate and resist to the immune system. A first CSC strategy to circumvent the immune attack is characterized by the low expression of both MHC molecules and antigen-processing machinery (APM), required for an efficient antigen presentation and necessary to stimulate T-cell activation or proliferation [30]. In a glioblastoma (GBM) model, the expression of MHC-I and -II, APM molecules and ligands of NKG2D (MHC class I–related chains A and B (MICA/B), UL16 binding proteins (ULBPs)) have been reported down-regulated or defective in CSCs [31]. This example highlights that a suboptimal immunogenicity by CSCs results in low or impaired susceptibility to T cell mediated immune responses. This represents a mechanism of immune evasion that is shared with normal stem cells. [31,32]. Further, ABCB5+ melanoma CSCs preferentially inhibited IL-2–dependent T-cell activation in a CD86-dependent manner and induced CD4+CD25+FoxP3+ regulatory T cells (T-regs) [33]. Melanoma CSCs displayed lower levels of MHC-I (but not MHC-II) and melanoma-associated antigens (e.g., MART-1, ML-IAP, NY-ESO-1, MAGE-A), while displayed higher levels of co-stimulatory molecules CD86 and PD-L1, responsible to their immune-evasive capacity [33]. In other settings, the CSC associated antigen CD44 has been positively associated with PD-L1 expression in lung adenocarcinoma [34]. Prostate CSCs showed overexpression of immune-inhibitory factors (e.g., PDL2 and TGF-β) and low expression of many HLA molecules [35]. A recent study demonstrated that the inefficient response of prostate cancer to chemotherapy is mediated by CSC resistance to Docetaxel, with low expression of differentiation markers (PSA, CK18, CK19) and HLA-I antigens, but overexpression of the Notch and Hedgehog signaling components [36]. These HLA-I defective prostate cancer cells are highly tumorigenic and their abundance correlates with tumor aggressiveness and poor patient prognosis. An additional interesting mechanism has been described in GBM and demonstrated how CSCs can induce apoptosis of both intratumor naive and activated T cell through galectin-3 secretion, allowing CSC expansion [37]. CSCs isolated from distinct solid tumors can not only evade immune attacks but also suppress actively immune responses releasing cytokines and soluble immunosuppressive factors (e.g., TGF-b, IL-10, IL-4 and IL-13) [38,39,40]. Immunosuppressive factors can recruit suppressive immune cells such as Tregs and M2 type macrophages to the tumor, can affect the TME components and subsequently remodel the TME to establish an immuno-suppressive environment [22,38,41]. 

**Figure 1:**
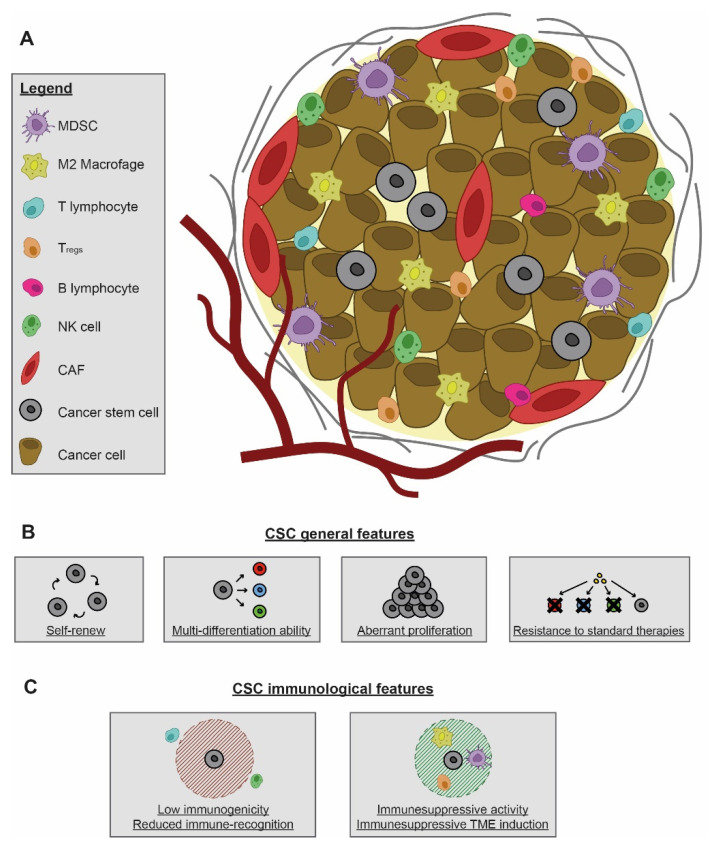
(**A**) Cellular components of the tumor microenvironment that shape tumor immunological landscape. The cellular part consists of immune cells of hematopoietic origin and stromal cells of non-hematopoietic origin. The immune cell compartment comprises tumor-infiltrating lymphocytes of T, B, and natural killer (NK) cells and tumor-associated myeloid populations of dendritic cells, macrophages, and myeloid-derived suppressor cells (MDSC). The stromal compartment consists of cancer-associated fibroblasts (CAF) and endothelial cells of blood and lymphatic vasculature. Cancer stem cells (CSCs) and immune components present in the tumor microenvironment exert the function of critical regulators of tumor growth. (**B**) Biological characteristics of CSCs. CSCs possess both self-renewal and multilineage differentiation abilities, leading to the composition of intratumoral heterogeneity. CSCs have aberrant proliferation and are responsible for resistance to anticancer treatments, including conventional chemotherapy, radiation therapy and molecularly targeted therapy. (**C**) Functional characteristics of CSCs. CSCs may present multiple strategies to circumvent the immune attack, including genetic and non-genetic alterations that allow reduced immune recognition, enhanced tolerance to cytotoxic effects of immunity and promotion of a protective immunosuppressive tumor microenvironment.

Cell surface molecules expressed on CSCs can also dampen immune responses. In breast cancer model, high levels of CD200 have been associated with the suppression of Th1 responses, decreased neutrophil infiltration and increased IL-10 production induced by the tumor [42,43]. PD-L1 is often over-expressed on tumor cells and PD-L1 up-regulation on CSCs is probably tumor-type or localization-dependent. Hypoxia, for example, is one of the triggers that can up-regulate PD-L1 with tumor glycolysis promoting function [44]. High expression of PD-L1 on CSCs has been reported on head and neck carcinoma [45], on CD133+ colorectal [46] and gastric CSCs [47], but not on melanoma CSCs [33]. In a recent study in squamous cell carcinoma, CD80 expressed on CSCs has shown higher affinity for CTLA4 than for CD28 on CD8+ cytotoxic T cells, dampening the effectiveness of cytotoxic T cells at attacking the tumor [48]. In GBM CSCs, immune-evasion can be due to high levels of MHC I and low levels of CD86 and CD40, but not MHC II or CD80 [37]. A summary of the main CSC features is reported in Figure 1.

### 2.2. Signaling Pathways’ Alterations in Cancer Stem Cells 

Several pathways playing a role in normal stem cells are frequently deregulated in CSCs: Myc, Notch, Hedgehog (Hh), Wnt, FGF/FGFR, EGF/EGFR, NF-κB, MAPK, PTEN/PI3K, HER2, and JAK/STAT [6,49,50,51]. Notch, Wnt/β-catenin, and Hh pathways are implicated in CSC regulation but are also responsible for immune cell behavior and peripheral effector function [52,53,54]. Notch signaling has been correlated to peripheral T-cell maturation into effector cells and cytokine production. Wnt/β-catenin pathway has a role in the regulation of T-cell development/activation and in the development of CD8+ memory T-cell. Hh signaling pathway is responsible for normal tissue homeostasis and development, including immune cell behavior and peripheral effector function [55,56,57,58]. As these pathways have multiple and physiological roles, targeting them is more challenging [59]. Furthermore, particular cells of the immune system play a complex role in CSC development. M2 macrophages can produce the immunosuppressive factors milk-fat globule EGF-8 (MFG-E8) and IL6. MFG-E8, in particular, promotes CSC resistance by activating Sonic Hedgehog signals and Stat3 pathway [60]. Moreover, it has been proposed that CSCs themselves can enter latency stage and escape natural killer (NK) cells killing by expressing DKK1, a WNT pathway inhibitor mechanism that allows the downregulation of the NK cell activation ligands [61]. Other pathways may govern immunological immunoresistance of CSCs in different cancer types: c-Myc upregulates the expression of the innate immune inhibitor CD47 and adaptive immune checkpoint molecule PD-L1 [62]. Loss of tumor suppressor PTEN leads to reduced expression of neoantigens responsible for immunoreactivity [63]. In the metastatic uterine leiomyosarcoma model, loss of PTEN is associated with resistance to anti-PD-1 checkpoint blockade therapy [63]. STAT3 signaling is constitutively activated in GBM CSCs and may have an immunosuppressive role, as inhibition of STAT3 can restore T-cell function [37]. Interesting recent data are linking the immunoresistance of CSC to their acquisition of an autophagic state, promoted by the stem-related gene NANOG through the hyperactivation of EGFR-AKT signaling [64].

### 2.3. CSCs Markers and Tumor Associated Antigens (TAA)

Most CSC markers have been identified based on the knowledge of stem cells in healthy tissues from which the tumors arise. Identification and isolation of putative CSCs have been established based on functional assays (e.g., Aldefluor, tumorspheres and organoids), side population (SP), staining of cell surface markers and fluorescence activated cell sorting (FACS). 

The first CSCs were identified within breast cancer. These CSCs were characterized by the expression of CD44 and low levels of CD24 [65]. 

Actually, a limited number of CSC markers have been reported, resulting promising targets for CSC immunotherapy [3,38,66,67,68,69] (e.g., CD44, CD133, HER2 and Prostate Stem Cell Antigen (PSCA)). It should also be considered that CSCs are characterized by plasticity and capacity to change their phenotypical and functional appearance, somehow limiting the relevance of precise individual markers. Additionally, most CSC markers, can be referred to heterogeneous subsets of CSC populations, highlighting that combinations of multiple markers may better contribute to a comprehensive CSC detection [13].

The CSC detection markers most commonly used across the variety of solid tumors are the following: CD133, CD44, IL-6R, CD24, epithelial cell adhesion molecule (EpCAM), leucine-rich repeat-containing G-protein coupled receptor 5 (Lgr5), CD166 and CD29, alone or in combination. Although for some of these markers is evident their stem cell function, their targeting may impair anti-tumor immune response. CD44 is a CSC marker in breast, prostate, colon, head and neck and pancreatic cancer [70], however CD44 also regulates T helper type 1 (Th1) cell survival and memory function [71], IL17 and IFN-γ production by T-cell [72]. IL-6 has been shown to enhance stemness markers (Oct-4, Notch, Lgr5) in colon cancer [73] and promote the survival and tumorigenicity of CSCs in head and neck carcinoma [74]. On the other side, IL-6R plays an important role in naive and central memory T-cells, regulating their survival, proliferation and effector function and blocking regulatory T-cell (Treg) function [75].

Additionally, a limited number of CSC markers are currently available but their biological function needs to be fully characterized, such as CSPG4 in multiple cancer types [76], EGFRvIII [77] and IL13Rα2 [78] in gliomas, and EpCAM in prostate cancer [79].

CSCs can express TAAs that potentially may be recognized by the immune system of the host [66]. Four different TAA subgroups have been described in CSCs [38,57,80,81,82]: (a) antigens (e.g., EGFRvIII, survivin, hTERT) highly expressed by the tumor but minimally expressed by normal tissues; (b) cancer/testis (CT) antigens (e.g., MAGE-A3, MAGE-A4, NY-ESO1) aberrantly present in tumor as they are normally expressed only by placenta and testicular germ cells; (c) neoantigens deriving from somatic mutations giving rise to new epitopes recognized by immune system; and (d) differentiation antigens (e.g., PSA in prostate cancer and MART-1 in melanoma) specific for a given tissue and expressed by both cancer and non-malignant cells. 

We acknowledge that most of these TAA are not exclusively expressed by CSCs but can be shared by non-CSC counterpart, providing opportunities to explore immunotherapy strategies targeting the CSC niche and strategies with a target broader than the CSC compartment. 

In this review we will explore studies either directly based on CSC-specific markers and based on CSC-shared TAAs.

## 3. Cellular Immunotherapy Targeting CSCs

Adoptive cell therapy is emerging as promising treatment for advanced cancers refractor to conventional treatments. Here we describe different killer lymphocyte populations, belonging either to the adaptive immune system or the innate immune response, with the potentiality to target CSCs in solid tumors. In the first group, we are summarizing some preclinical and clinical studies based on CSC-primed T cells, while in the second we are focusing on preclinical and clinical data regarding innate immune effectors (Natural Killer (NK) cells, Cytokine Induced Killer (CIK) cells and γδ T cells). The immunotherapy field was recently boosted with the emergence of chimeric antigen receptor (CAR) T cells therapy [83]. Since their antigen-recognizing receptor is based on modified antibodies, CARs can specifically target surface antigen, such as the CD19 co-receptor on B cells [84]. The success of CAR-T therapies in hematological malignancies has given rise to hope of extending the use of this strategy to further cancer indications, including solid tumors, especially considering the proportion of new cases of patients with solid tumors per year as compared with hematological tumors [85]. For each different type of antitumor killer lymphocyte considerable for adoptive cell therapies, we will also report the initial data available about their possible engineering with CARs. 

Advances in immunotherapy and the development of CAR strategy have provided a solid and successful approach to target membrane protein expressed by cancer cells. CARs are synthetic receptors composed by an extracellular domain based on the single chain variable fragment (scFv) derived from a tumor antigen-specific monoclonal antibody (mAb) fused into TCR-derived signaling domain and with one or more costimulatory domains [86,87]. Impressive therapeutic efficacy of CAR-mediated cell therapy has been observed in a series of clinical trials, especially those for chronic lymphocytic leukemia and acute lymphoblastic leukemia [88]. 

The choice of tumor antigens restrictively expressed on the surface of malignant cells is important in CAR-mediated cell therapy to target cancer cells or even CSCs. CSCs abnormally express stemness-associated genes, some of which play vital roles in embryonic development. These genes may serve as potential targets, as they are expressed at high levels on the membrane of tumor cells, especially CSCs, but are scarcely expressed in normal tissues. Identification of CSC-specific TAA is crucial to target the novel CSC subset, responsible for tumor maintenance and recurrence. 

### 3.1. T Cell-Based Strategies Targeting CSC

Adoptive T cell therapy requires the generation and expansion of effector T cells followed by their infusion back into patients. The efficient conventional targeting of CSCs by T cells depends upon a sufficient level of HLA class-I molecule expression and intact antigen presenting machinery in these cells. Two main strategies have been developed to generate CSC-specific T cells: CSC-primed T cells and CAR-engineered T cells. 

In the first case, T lymphocytes are generally stimulated and primed in vitro by CSC lysate-pulsed or peptide-pulsed autologous dendritic cells (DCs). In a preclinical study using ALDH1A1 peptide-pulsed autologous DCs, CSC-specific CD8+ T cells were generated and transferred in xenograft mice of squamous cell carcinoma of the head and neck (SCCHN). CSC-specific CD8+ T cells eliminated ALDH1A1^bright^ CSCs, inhibited tumor growth and metastases, and prolonged the survival in the treated cohort [89,90]. Similarly, in a colorectal cancer study CD8+ T cells, repeatedly stimulated with autologous PHA-blasts pulsed with the ASB4 CSC specific peptide, were adoptively transferred in a mouse model effectively preventing tumor growth [91]. In a lung cancer study, CSCs^ALDHhi^ were isolated and their lysate-pulsed DCs used to stimulate CD8+ T cells. Subsequently, these ALDH^high^-CD8+ T cells exhibited significant antitumor effects, resulting in inhibition of tumor growth and extended survival [92]. In bone malignant fibrous histiocytoma (MFH), a CTL clone was induced by mixed lymphocyte tumor cell culture using autologous peripheral blood mononuclear cells and freshly isolated SP cells, consequently this clone showed specific cytotoxicity against SP cells [92].

Problems in targeting CSCs with primed T cells are the immune escape of tumors caused by antigen loss and that antigens recognized by CSC-primed T cells remain largely unknown. Furthermore, CSCs are often poorly targeted by T cells because of a lower MHC class I expression and a higher production of IL4 than the non-CSC counterpart, as observed in colon CSCs [31,93]. 

CSCs can share TAAs expression with the non-CSC counterpart or can express TAAs that are functionally linked to cancer stemness. These latter TAAs may result in more clinically relevant successes [94,95,96]. Models encompassing T cells engineered with CARs against the CSC antigens have been developed and studied in different solid tumor settings. To date, a limited number of reports, mostly in animal models, have been published on CSC targeting by CAR T cells. The pre-clinical and clinical trials as well as the most attractive markers for targeting by CAR T cells are discussed below. 

In preclinical models, CAR T cells have been designed to target CSC-associated antigens, such as CD133 in glioblastoma [97], CSPG4 in multiple cancer types [79], EGFRvIII [80], IL13Rα2 [81] and EphA2 in gliomas [98,99], SSEA1 in medulloblastoma, glioblastoma and neuroendocrine tumors [100], HER2 in osteosarcoma [101], GD2 and TEM8 in breast tumor [102,103], EpCAM and PSCA in prostate cancer [82,104]. These studies have demonstrated the antitumor effects of CAR T cells by targeting CSCs and suggest that CSC-specific T lymphocytes can be generated, in vitro expanded and adoptive transferred into tumor-bearing hosts to target CSCs in order to eradicate or to control tumor growth in vivo. A further study using anti-EpCAM CAR T cells for local treatment of peritoneal carcinomatosis in xenograft mice demonstrated the efficacy of this approach for the treatment of gastrointestinal and gynecologic malignancies [105]. Numerous preclinical studies have indicated other surface markers potentially useful to identify or target CSCs: CD90, ALDH, CD47, CD44, CD24, microtubule-associated doublecortin-like kinase 1 (DCLK1) that are expressed in multiple cancer types with a higher expression in CSCs compared to other bulk tumor cells [82,97,106,107]. DCLK1 has been described as a CSC associated antigen in colon, pancreatic [108,109,110] and even in Cholangiocarcinoma (CCA) tumors [111]. Recently, preclinical findings supported promising results with adoptive immunotherapy based on DCLK1-CAR T lymphocytes against colorectal cancer (CRC) [107].

In prostate cancer, preclinical studies with PSCA CAR T cells demonstrated that PSCA is a promising target for immunotherapy of prostate cancer [112,113]. Bispecific antibodies targeting PSCA/PSMA have been developed to increase “tumor-sensing” and reduce potentially harmful reactivity against healthy tissues expressing either antigen alone [114,115]. A limited number of clinical trials concerning CAR T cells targeting antigens associated to CSCs are registered on the website www.clinicaltrials.gov (accessed on 1 January 2021).

Two distinct clinical trials using anti-EGFR^VIII^ CAR T cells in patients with EGFR^VIII+^ recurrent GBM were not successful: no appreciable tumor regression and no objective responses have been reported in any patients enrolled, probably for the high heterogeneity of EGFR^VIII^ expression and for the tumor immunosuppressive microenvironment [116,117]. Encouraging results are reported in other clinical studies. A case report on a patient with advanced cholangiocarcinoma treated with anti-EGFR CAR T cells combined with anti-CD133 CAR T cells indicated the feasibility of clinical cancer treatment with CSC-targeted CAR T cells. EGFR-CAR T cells infusion showed partial response of 8.5 months and extra 4.5 months upon receiving CD133-CAR T cells, with some degree of toxicity [118]. Local infusions of IL13Rα2-specific CAR T cells into a patient with recurrent GBM caused regression of the primary and metastatic tumors for 7.5 months without toxic effects. Subsequently, the patient develops tumor at several new locations, this might be due to the lower expression of IL13Rα2 in the new sites [119]. A phase I trial tested CD133-directed CAR T cells for advanced metastasis malignancies (HCC, pancreatic, and colorectal cancers): this study reported outcomes between partial remission and stable disease with controlled toxicity [120]. Utilizing well-characterized CSC markers, it is possible therefore to use CAR T cells to eliminate CSCs in many cancers. CAR T cells alone or in combination with standard therapies or checkpoint inhibitors are a promising strategy for the treatment of many cancers. Currently, the majority of clinical trials based on CAR-T cell therapy directed against CSCs are ongoing.

### 3.2. Innate Immune Effectors Targeting CSCs

#### 3.2.1. NK Cells

Natural killer (NK) cells are large granular lymphocytes, constitute 5–15% of circulating lymphocytes, and represent the main effectors belonging to the innate immunity [121]. NK cells recognize tumor cells and infected cells, in a HLA independent manner without prior sensitization [122] and release pre-formed granules known as perforin and granzyme B, which can induce necrotic as well as apoptotic or programmed cell death in target cells [123,124,125]. NK cells simultaneously express activating and inhibitory receptors that encounter target cells by the subtle balance of transmitted signals for activation or inhibition [126]. NK cells mediate direct and antibody-dependent cellular cytotoxicity (ADCC) against tumors and regulate the function of other cells through the secretion of cytokines and chemokines [127]. 

Adoptive NK cell therapy has been explored with either autologous or allogenic NK cells [128,129]. Ex vivo NK cell culture is demanding because of their limited life span and expansion potential [130]. Established human NK cell lines (e.g., NK92, KHYG-1, NKL and NKG) can also be explored as a valuable alternative to primary NK cells. NK92 cell line in particular is approved by the US Food and Drug Administration (FDA) for phase I and II clinical trials, allowing a “off-the-shelf” CAR.NK92 production [131,132]. NK92 can easily be expanded to high numbers and maintained for therapeutic use in the presence of IL2, while retaining consistent phenotypic and functional features [133]. 

Increasing data demonstrate that NK cells can selectively identify and lyse CSCs [134,135], as they have low or no MHC-I molecules but up-regulate the ligands for NKG2D, DNAM1 and NKp30 NK-activating receptors [134,136,137]. Several studies highlighted NK cell ability to recognize and kill poorly differentiated tumors [138,139,140] and that cytokine-activated (IL2 and/or IL15-activated) NK cells were effective against human breast, colon, melanoma and glioblastoma CSCs [141,142,143]. Studies on oral squamous carcinoma reported higher levels of NK cell activating ligands on CSCs as compared to non-CSCs, resulting in their higher sensitivities to NK cell killing [144]. In particular, Tallerico and colleagues observed lower levels of MHC class I expression on colorectal cancer CSCs compared to non-CSCs. They demonstrated that CSCs showed increased susceptibility to NK killing [145], linked to upregulation of the activating natural cytotoxicity receptors, particularly NKp30 and NKp44. Castriconi and colleagues demonstrated low or absent expression of MHC class I molecules on GBM-derived CSCs and their high susceptibility to both allogeneic and autologous NK cells in co-culture models after pre-treatment with IL-2 and IL-15 [141]. In melanoma, Pietra and colleagues reported that both CSCs and non-CSCs showed sensitivity to activated allogeneic NK cells, possibly mediated by the DNAM-1 ligands Nestin-2 and PVR [146]. In breast cancer, Yin and colleagues reported that CSCs showed to be lysed by IL-2 and IL-15 activated NK cells, and such cytotoxicity was likely mediated by the increased expression of NKG2D ligands ULBP1, ULBP2 and MICA on CD44^+^CD24^−^ CSCs [143]. On the other side, CSCs can evade NK cell killing by shedding MICA and MICB and also by recruiting immunosuppressive Treg cells [147,148]. In glioma, CD133+ brain CSCs do not express either detectable MHC-I and NK cell activating ligands, escaping NK cell-mediated-immune surveillance [149]. IFN-γ stimulated the expression of these molecules on CD133+ CSCs, restoring their sensitivity to NK cell-mediated lysis in vitro [149]. In a preclinical study high-grade non-muscle invasive bladder cancer (NMIBC), NK cells from healthy donors, but not from NMIBC patients, upon activation with IL-2 and IL-15, could kill both CSCs and bulk tumor cells, promoting their differentiation and enhancing the efficacy of a possible combined chemotherapy [150]. In oral squamous carcinoma, a preclinical study proposed a novel NK ex vivo expansion and activation strategy, based on co-culture with osteoclasts to generate “super-charged” NK cells endowed with higher secretion of IL12 and IL15, increasing killing capability against CSCs [151].

Evasion mechanisms induced by TME can block NK cell mediated-CSC lysis through an increase in IL6 and IL8 secretion and decrease in IFNγ secretion. NK cells can reach a functional state, known as “split anergy”, characterized by a reduced NK cell cytotoxicity maintaining cytokine and chemokine secretion. This NK functional state decrease NK cytotoxic activity against CSCs but induce CSCs differentiation trough cytokine production, especially IFNγ and TNFα [152]. This phenomenon was found to be associated with an increase in MHC class I, PD-L1, and CD54 expressions and a reduction in CD44 levels on tumor cells [153].

NK cells are a promising approach to target both CSCs and non-CSCs, leading to prolonged therapeutic results. NK cells targeting of CSCs may initiate and amplify adaptive T cell-mediated responses. Previous clinical trials using NK cells as monotherapy in solid tumors obtained modest results, new trials point to a new application of NK cells in combination with traditional treatments in order to overcome the therapeutic resistance which CSCs may contribute [147].

Different promising strategies are based on antibody anti-CSC markers, such as CD44, CD24, CD133 and ALDH-1 [154] and bispecific antibody concomitantly binding CD16 on NK cells and CD133 on colorectal CSCs, that significantly improved CSC targeting ability by NK cells [155]. Finally, NK cells administration and concomitant inhibitory killer immunoglobulin receptor (KIR)-blockade, with or without other cancer drugs, may represent new opportunities for cancer patients [156]. CAR engineering of NK cells can enhance their specific recognition and elimination of tumor cells, providing an opportunity to generate NK-cell therapeutics of defined specificity. Numerous preclinical studies demonstrated the successfully generation of CAR-NK cells [157,158]. Furthermore, several groups improved CAR-NK activity including in the receptor construction one or more signaling domains derived from CD244 (2B4), NKG2D, DAP10 or DAP12 [157,159,160]. CAR.NK cell therapy presents several advantages compared to CAR-T cells: a) reduced on-target/off-tumor toxicity and low cytokine storm risk as they have limited in vivo persistence b) CAR-NK cells, endowed with innate killing activity, can attack tumors with heterogeneous expression of the CAR target antigen [161]. Preclinical study demonstrated that CAR-NK cells targeting specific antigens linked to CSCs (e.g., GD2, EGFRvIII, ErbB2, CD133, PSCA) displayed superior anti-tumor activity compared to parallel-unmodified NK cells. CAR-NK cells against prostate stem cell antigen (PSCA) displayed in vitro anti-tumor efficacy against PSCA^+^ CSCs, and GD2-CAR.NK showed cytotoxic activity against neuroblastoma and Ewing sarcoma cells [162]. GD2-CAR.NK92 cells have been tested in preclinical assays against neuroblastoma, melanoma, breast carcinoma and Ewing sarcoma, demonstrating selective anti-tumor activity [160,162,163,164]. Different preclinical approaches employ CAR-NK cells for GBM immunotherapy [165]. Stem-like GBM cells seem to be more sensitive to natural cytotoxicity of NK cells, as CSCs showed increased expression of ligands for activating NK cell receptors and down-regulated class I HLA ligands for NK cell inhibitory receptors [166] Preclinical data with NK92 cells showed that ErbB2-CAR.NK92 cells lysed ErbB2-positive stem-like GBM cells growing as neurospheres quite rapidly. EGFRvIII-CAR.NK92 inhibited tumor growth and extended survival of GBM xenograft. Other studies highlighted that ErbB2-CAR.NK92 prolonged survival and induced reduction of primary tumors and metastasis in breast cancer and GBM xenografts, while parallel-unmodified NK92 cells were unable to inhibit tumor progression [159,167,168]. CD133-CAR.NK92 have been explored in vitro against GBM and ovarian cancer in combination with cisplatin, demonstrating efficient anti-tumor activity [169]. A phase I clinical trial (CAR2BRAIN, protocol number NCT03383978) based on intracranial injection of ErbB2-CAR.NK92 in patients with recurrent ErbB2+ GBM is currently ongoing [168,170,171].

#### 3.2.2. CIK and NKT Cells

CIK cells are heterogeneous ex vivo lymphocytes featuring a mixed T- and NK cell phenotype, generated and expanded in vitro from peripheral blood mononuclear cells (PBMC) and endowed with MHC-independent antitumor activity [172,173,174,175,176,177]. CIK cells can be easily and efficiently expanded with the timed addition of IFN-γ, antibody (Ab) anti-CD3 and IL2 [178,179]. At the end of the expansion, the CD3+CD56+ cells represent the subset with the most potent cytotoxic activity against multiple tumor types [180,181]. The cytotoxic activity is primarily mediated by the interaction between the activating natural killer cell receptors of CIK cells, in particular NKG2D, and the corresponding stress-inducible ligands, including MIC A/B and ULBPs [182,183,184]. The clinical activity and safety profile of CIK cells was demonstrated in several clinical trials in both hematological and solid settings [175,185,186,187,188,189].

Immunotherapy based on CIK cells may overcome limitations caused by tumor downregulation of MHC molecules on CSCs and may be advantageous over T cells endowed with MHC-dependent activity. Further, CIK cells may be a valuable therapeutic strategy applicable to all patients, regardless their HLA-haplotypes.

Patient-derived CIK cells showed a potent killing ability against CSCs in preclinical in vitro and in vivo studies against putative melanoma, sarcoma, hepatocellular carcinoma (HCC) and nasopharyngeal carcinoma (NPC) CSCs [190], with possible relevant clinical implications. In these studies, CIK cells were equally effective against both putative CSCs and non-CSCs. In distinct studies putative CSCs were visualized with a promoter-fluorescence reporter gene strategy, where cancer cells were transduced with a lentiviral “CSC-detector” with GFP gene under control of the stem cell-specific Oct4 or Nanog promoter [191,192,193]. Based on this methodology, CIK cells showed to be effective to kill CSCs surviving to chemotherapy or targeted therapy either in in vitro and in vivo preclinical studies.

In another investigation, CIK cells sensitized by EpCAM and CD44 peptide DCs were effective in vitro against Prostate Cancer Stem like Cell (PCSC)-enriched prostate-spheroids and in vivo against PCSC-enriched prostate-spheroid xenografts [194].

Recent evidences showed the success of CIK cell redirection by CAR strategy to enhance CIK cell anti-tumor efficacy [26,195,196,197,198,199]. CAR.CIK are an appealing platform for CAR engineering, as they may generate bipotential killers combining the specificity of CAR with their intrinsic tumor killing capacity [200,201].

In recent years, preclinical studies reported first evidences of enhanced CAR.CIK activity in high grade soft tissue sarcoma (STS) thanks to the expression of a CAR specific for CD44v6 antigen or CSPG4 [26,27], and in NPC thanks to a CAR specific for 5T4 antigen [202]. These CAR.CIK cells could efficiently eliminate tumor cells and also stem cell-like cells in vitro, as these tumor antigens are shared with CSCs and involved in tumor initiating process, EMT and clinical aggressiveness. In addition, a first evidence showed in vitro the anti-tumor efficacy of CIK cells expressing a CAR specific for CSPG4 antigen in high grade STS [27]. It has been widely described that CSPG4 has a key role in several oncogenic pathways required for malignant progression and metastatization and is overexpressed by tumor cells and CSCs [203]. Overall CAR-CIK cells have gradually become a realistic new option of cancer immunotherapy and are studying in vitro and in vivo as a potential effective platform against a wide variety of cancers. Further preclinical and clinical investigation are needed to evaluate the potential of targeting putative CSCs with CIK cells, also in synergism with other therapeutic strategies.

Natural killer T (NKT) lymphocytes respond rapidly to a wide variety of glycolipids and stress-related proteins and share properties of both T and NK cells, such as CD56, CD16 expression and granzyme and perforin productions [204,205]. In contrast to CIK cells, they are already present in small percentage in blood circulation; NKT cells express an invariant αβTCR that recognizes antigens presented by MHC class I CD1d molecule, as glycolipids. NKT cells are involved in anti-tumor immunity acting as recruiter of adaptive immune cells trough their rapid cytokine secretion [206]. Due to their restriction to the monomorphic HLA-like molecule CD1d, but not to HLA, NKT CAR cells show potential for enabling off-the-shelf cancer immunotherapy, even if dedicated clinical trials have not yet been reported. NKT cells may be isolated from patients or allogenic donor and are most commonly expanded with the glycolipid α-GalCer, transduced to express a tumor-specific CAR and reinfused in cancer patients with a favourable safety profile based on absence of alloreactivity and limited in vivo persistence [207,208].

Few preclinical studies reporting the cytotoxic activity of NKT cells against CSCs are based on redirection with CARs. In neuroblastoma and B cell lymphoma, GD2-CAR.NKT cells efficiently localized at tumor site, reduced tumor growth and prolonged survival of xenograft models, targeting also GD2^+^ CSCs [208]. CSPG4-CAR.NKT cells displayed similar efficient cytotoxicity compared to conventional CAR.T cells redirected by CSPG4-CAR [209].

Currently, an ongoing phase I clinical trial is exploring efficacy and persistence of autologous GD2-CAR.NKT cells in neuroblastoma patients (GINAKIT, NCT03294954).

#### 3.2.3. γδ T Cells

γδ T lymphocytes are unconventional non-MHC-restricted T cells, characterized by an invariant γδTCR. They represent 1–5% of circulating lymphocytes and are a significant subset of resident T cells in lymphoid organs, epidermis, gastrointestinal mucosa, and reproductive system [210]. The Vγ9Vδ2 phenotype is the most represented of peripheral blood γδ T lymphocytes, while δ1 e δ3 are mostly tissue-located [211,212,213]. Γδ T cells recognize stress inducible molecules and are characterized by their ability to recognize early metabolic changes, recognizing non-peptide metabolites like phosphoantigens or aminobisphosphoantigens and the cholesterol precursor isopentyl pyrophosphate, that differentiate healthy cells from transforming ones [214]. γδ T cell protection against cancer occurs mainly by the production of pro-inflammatory cytokines such as IFNγ, TNFα, and IL-17 and through their cytotoxic activity [215].

In preclinical studies, Vγ9/Vδ2 T cells efficiently killed CSCs derived from colon cancer [216], ovarian cancer [217], and neuroblastoma [218] but were less effective against prostatic CSCs [219] and breast cancer [220]. CSCs derived by breast cancer showed to be hypo-responsive to γδ T-cell targeting. Breast CSCs are characterized by increased levels of PD-L1, anti-apoptotic MCL-1 and MICA shedding compared with non-stem counterpart. In vitro either PD-1 blockade or treatment with MCL-1 degrader or proteolytic cleavage inhibitor (ADAMi, GW280264X) were able to restore breast CSCs sensitivity to γδ T-cell cytotoxicity [220]. In breast cancer, γδ T cells could kill CSCs, expressing relatively low levels of MHC-I and CD54, following pre-treatment with γδ T-cell agonist zoledronate. Zoledronate exposure increased γδ T cells proliferation rate, TNFα and IFNγ secretion, granzymes production, expression of CD69 molecule and tissue-homing chemokine receptors CCR5 and CXCR3, while decreased lymphoid-homing chemokine receptors CCR7 and CXCR5 [216,218]. Zoledronate-activated γδ T-cells enhanced the killing activity of CD8+ T cells through the IFN-γ-mediated upregulation of MHC-I and ICAM-1 molecules [217]. Combination therapy with γδ T cells and zoledronate is feasible in patients with different advanced solid tumors [221].

However, clinical trials stimulating γδ T cells or even transferring γδ T cells with or without activating stimuli into cancer patients show very low efficiency and very limited success [222,223,224]. This might be due to the lack of knowledge regarding the specificity and diversity of these cells. In breast cancer, synergism between CD8+ T cells and γδ T cells has been described in the eradication of tumor cells including CSCs: γδ T cells induced upregulation of MHC class I and CD54/ICAM-1 on CSCs, enhancing their susceptibility to CD8+ T cells [225]. γδ T cells are expected to be associated with the same level of safety reported for CAR-NK cells [157] and may represent an intriguing T cell subset to exploit CAR redirection as a possible strategy to target CSCs.

An overview of the main cellular immunotherapy clinical trials directed against CSC-relevant targets is reported in Table 1.

## 4. Conclusions and Challenging Perspective

Biological and immunological characterization of CSCs as long as definition of their interaction with immune cells in the TME are crucial to set up more efficacious strategies and innovative anticancer therapies. New emerging methodologies, as single cell molecular analysis, may provide new insights in understanding the relationship among immune characters, stromal, cancer cells and CSCs in the TME and elucidate their heterogeneous contribution in tumor progression [226,227].

Important challenges need to be faced to develop new and more effective immunotherapy strategies capable to involve CSCs. The first is represented by the heterogeneity in CSC populations. Distinct CSC subpopulations expressing different phenotypic markers have been reported inside the same cancer type [3]. This means that a specific immunological treatment could eliminate only a subset of CSC. Furthermore, CSCs may escape from antigen-dependent immunotherapies by lacking or decreasing the target density on their surfaces. In addition, the majority of the reported CSC markers and TAAs are not CSC-exclusive, and therefore identification of CSC-specific antigens is critical for the success of antigen-dependent immunotherapies, avoiding potential toxicities and achieving treatment specificity. For instance, in CAR based immunotherapies, one of the major difficulty is the possible development of on-target/off-tumor toxicity caused by CAR cell killing activity against normal cells [106].

The second important challenge is tumor cell plasticity. CSCs constantly evolve as well as tumor evolves and progresses, further CSCs evolve upon treatment. Tumor plasticity represents a huge hurdle in the development of durable targeted cancer therapies, as eradication of existing CSC populations might be followed by their regeneration, under treatment pressure, from non-CSC counterpart within the tumor [3]. Promising results may derive from MHC-unrestricted approaches (e.g., NK and CIK cells), as they kill without HLA restrictions and might recognize ligands whose expression is induced by heterogeneous stimuli as stress, chemotherapies and other agents, overcoming issues arising from tumor plasticity and heterogeneity.

Another challenge is the CSC low immunogenicity and negative immunomodulating effects. CSCs are mainly resistant to conventional cancer therapies, as they can escape from antitumor immunity through lower expression of antigens and HLA recognized by immune cells.

Lower immunogenicity of CSCs may be enhanced by inhibiting negative immunoregulatory pathways and by upregulating HLA I and APM components through combination therapies with IFNs, chemotherapy, radiotherapy, and/or epigenetic treatments [38]. A new intriguing possibility is represented by epigenetic therapies combined with immunotherapy, as epigenetic drugs modulate the expression of immune-related genes either on tumor cells and on tumor-associated immune cells [228].

All the reported CSC features may have contributed to the disappointing outcomes of current adoptive immunotherapies in solid tumors. Strategies that combine conventional anti-tumor therapies and CSC-specific immunotherapies would be desirable to eradicate cancer.

In the future, CAR effector cells specific to CSCs combined with chemotherapy, radiotherapy or immune checkpoint inhibitors will hopefully be more effective, helping to achieve better outcomes as compared to monotherapies.

Immunotherapy strategies based on NK and CIK cells have the advantage over other types of autologous T cell therapies, including CAR T cells, of an intrinsic tumor-killing ability by recognizing HLA-independent inducible stress ligands [229]. These properties extend their therapeutic value to numerous types of solid tumors. Applying CARs or bispecific antibodies to NK and CIK cells, we could hopefully add specificity to their tumor killing capabilities [155,198,199,230,231].

In near future, rigorous evaluation of the different cell therapy strategies alone or in combination with other treatments (e.g., chemotherapy and/or radiotherapy) is advisable to provide insights into the optimization and development of novel anti-cancer immunotherapy protocols capable of involving CSCs.

## Figures and Tables

**Table 1:** Adoptive immunotherapy trials involving CSC-relevant targets.

Biological Agent	Strategy	Combination	Disease Target	NCT Identifier	Status	Phase
NK-cell based therapy	**HER2-CAR.NK-92** (CAR2BRAIN)	/	Recurrent Glioblastoma	NCT03383978	Recruiting	1
NKT-cell based therapy	**GD2-CAR.NKT** (GINAKIT)	Cyclophosphamide Fludarabine	Neuroblastoma	NCT03294954	Recruiting	1
T-cell based therapy	**IL13Rα2-CAR.T**	/	Refractory Malignant Glioma	NCT02208362	Recruiting	1
	**CD133-CAR.T**	/	Liver Cancer Pancreatic Cancer Colorectal Cancer Brain Tumors Ovarian Cancer Breast Cancer	NCT02541370	Completed	1–2
	**EGFRvIII-CAR.T**	Aldesleukin Cyclophosphamide Fludarabine	Malignant Glioma Glioblastoma Gliosarcoma	NCT01454596	Completed	1–2
	**EGFRvIII-CAR.T**	/	Recurrent Glioma	NCT02209376	Terminated	1
	**EGFR-CAR.T** plus **CD133-CAR.T**	/	Cholangiocarcinoma	/	Case Report	/
	**MUC1-CAR.T PD-1 KO**	/	Advanced Esophageal Cancer	NCT03706326	Recruiting	1–2
	**EGFR/IL-12-CAR.T**	/	Metastatic Colorectal Cancer	NCT03542799	Not Yet Recruiting	1
	**MESO-CAR.T**	/	Refractory Relapsed Ovarian Cancer	NCT03916679	Recruiting	1–2
	**MESO-19-CAR.T**	/	Metastatic Pancreatic Cancer	NCT02465983	Completed	1
	**EpCAM-CAR.T**	/	Recurrent Breast Cancer	NCT02915445	Recruiting	1
	**LeY-CAR.T**	/	Advanced Cancer	NCT03851146	Recruiting	1
	**MOV19.BBz-CAR.T**	/	Recurrent High-grade Serous Ovarian Cancer	NCT03585764	Recruiting	1
	**PSCA-CAR.T**	Cyclophosphamide Fludarabine Fludarabine Phosfate	Castration-Resistant Prostate Carcinoma Metastatic Prostate Carcinoma Stage IV Prostate Cancer	NCT03873805	Recruiting	1

## Data Availability

Not applicable.
